# The burden of cervical pre-cancer and cancer in HIV positive women in Zambia: a modeling study

**DOI:** 10.1186/s12885-015-1558-5

**Published:** 2015-07-24

**Authors:** Allen C. Bateman, Katundu Katundu, Mulindi H. Mwanahamuntu, Sharon Kapambwe, Vikrant V. Sahasrabuddhe, Michael L. Hicks, Benjamin H. Chi, Jeffrey S. A. Stringer, Groesbeck P. Parham, Carla J. Chibwesha

**Affiliations:** 1 Centre for Infectious Disease Research in Zambia, Lusaka, Zambia; 2 University of North Carolina at Chapel Hill, Chapel Hill, NC USA; 3 University of Zambia, Lusaka, Zambia; 4 Vanderbilt University, Nashville, Tennessee, USA; 5 Michigan Cancer Institute, Pontiac, MI USA

**Keywords:** Cervical cancer, Cervical intraepithelial neoplasia, HIV, Zambia

## Abstract

**Background:**

HIV infection is associated with a higher incidence of precancerous cervical lesions and their progression to invasive cervical cancer (ICC). Zambia is a global epicenter of HIV and ICC, yet the overall burden of cervical pre-cancer [cervical intraepithelial neoplasia 3 (CIN3)] and ICC among its HIV positive adult female population is unknown. The objective of this study was to determine the burden of cervical disease among HIV positive women in Zambia by estimating the number with CIN3 and ICC.

**Methods:**

We conducted a cross-sectional study among 309 HIV positive women attending screening in Lusaka (Zambia’s most populated province) to measure the cervical disease burden by visual inspection with acetic acid enhanced by digital cervicography (DC), cytology, and histology. We then used estimates of the prevalence of CIN3 and ICC from the cross-sectional study and Spectrum model-based estimates for HIV infection among Zambian women to estimate the burden of CIN3 and ICC among HIV positive women nationally.

**Results:**

Over half (52 %) of the study participants screened positive by DC, while 45 % had cytologic evidence of high grade squamous intraepithelial lesions (SIL) or worse. Histopathologic evaluation revealed that 20 % of women had evidence of CIN2 or worse, 11 % had CIN3 or worse, and 2 % had ICC. Using the Spectrum model, we therefore estimate that 34,051 HIV positive women in Zambia have CIN3 and 7,297 have ICC.

**Conclusions:**

The DC, cytology, and histology results revealed a large cervical disease burden in this previously unscreened HIV positive population. This very large burden indicates that continued scale-up of cervical cancer screening and treatment is urgently needed.

## Background

Invasive cervical cancer (ICC) is among the most common cancers worldwide, accounting for an estimated 270,000 deaths annually [1]. Cervical cancer is the most common cause of cancer death among women in sub-Saharan Africa [2], and Zambia has the second-highest incidence rate of ICC in the world [1]. Sub-Saharan African countries are also home to the majority of HIV infections, accounting for 71 % of the people living with HIV worldwide [3]. In Zambia, the HIV prevalence in adults was estimated at 13 % in 2012 [3], and there are an estimated 375,000 women aged 20–44 with HIV. HIV compounds the ICC burden, because HIV is associated with a higher prevalence of persistent infection with high-risk human papillomavirus (HPV) – an obligate cause of ICC [4–6]. HIV infection is also associated with a higher incidence of precancerous cervical lesions and accelerated progression of these lesions to ICC [7]. The effect of antiretroviral therapy (ART) on the natural history of cervical precancerous lesions is unclear [7], with some studies showing no effect and other studies showing a modest beneficial effect of ART [8, 9].

The prevalence of cervical pre-cancer and cancer is reported to be high among HIV positive women in sub-Saharan Africa. The prevalence of cervical squamous intraepithelial lesions (SIL) among HIV positive women in Nigeria was 13 % [10], SIL was detected in 43.5 % of HIV positive women initiating ART in Cameroon [11], and pre-cancerous lesions (CIN1 or worse) were detected in 27 % of HIV positive women attending an ART clinic in Kenya [12]. A study among HIV positive women initiating ART in South Africa reported that 66 % of women had an abnormal Pap smear, including 40 % with LSIL and 10 % with HSIL [13]. A 2005 report from Zambia by this group described the prevalence of SIL in HIV positive women, and found a prevalence of 53 % for high-grade SIL and worse, one of the highest reported in any population [14]. However, the overall disease burden of histologically-proven cervical pre-cancer [(cervical intraepithelial neoplasia 3 (CIN3)] and ICC in HIV positive women in Zambia is unknown.

Herein we extend our initial findings [14] by conducting a cross-sectional study of twice as many HIV positive women in Lusaka as the 2005 study, and assess cervical disease by DC, cytology, and histology. We compared the women in the cross-sectional study with women enrolled in the Cervical Cancer Prevention Program in Zambia, and used the prevalence of CIN3 and ICC from the cross-sectional study to estimate the disease burden of CIN3 and ICC among HIV positive women nationally in Zambia. The objective of this study was to provide modeled estimates of the number of HIV positive women in Zambia with CIN3 and ICC.

## Methods

### Cervical Cancer Prevention Program in Zambia

In response to the high burden of cervical SIL in HIV positive women in Lusaka that was found in initial studies [14], the Cervical Cancer Prevention Program in Zambia (CCPPZ) was established in 2006. CCPPZ provides services through the Zambian public sector platform and is integrated into the existing HIV/AIDS care and treatment infrastructure. The program offers visual inspection with acetic acid enhanced by digital cervicography (DC) with same-day cryotherapy or cold coagulation for eligible precancerous lesions, or referral for loop electrosurgical excision procedure (LEEP) or punch biopsy for ineligible lesions [15–17]. CCPPZ originally provided services to HIV positive women, but in response to overwhelming demand, services were made available to all women by mid-2006, regardless of HIV status. To date, CCPPZ has screened more than 180,000 women and has scaled up services to all provinces in Zambia. The CCPPZ clinical database includes the years 2006–2013 and contains individual-level information on women screened at 12 public clinics in the Lusaka area. Data is entered by nurses into Access databases at the time of screening and is maintained by the CCPPZ program.

### Cross-sectional study

Between February 2008 and December 2011, we enrolled 309 HIV positive women aged 20–45 into a cross-sectional screening study at Matero public health clinic in Lusaka, Zambia. This clinic serves the Matero community, and approximately 1500 women at the clinic are screened for cervical cancer each year through CCPPZ. During the study period, women were identified in the HIV Care and Treatment clinic, where they were made aware of cervical cancer screening and invited to attend the cervical cancer prevention clinic. All HIV positive women attending the clinic were invited to participate in the study. At the cervical cancer prevention clinic, a nurse provider counseled patients on cervical cancer screening and the research protocol for this project, and obtained informed consent from women willing to participate in the study. The inclusion criteria were being HIV positive, non-pregnant by self-report, between 20–45 years of age, and healthy enough to undergo a pelvic examination. Following written informed consent, demographic information was collected. ART status was by self-report: ART-naïve was defined as never having received ART, while ART-experienced women were either currently or previously on ART. This was the first cervical cancer screening visit for each woman. All women were then screened with cytology and DC as part of a cross-sectional study to compare the test characteristics of cytology to DC [18]. Trained, experienced nurses performed all study procedures.

Pap smears were obtained using a wire brush for sampling of the endocervix, and an Ayres plastic spatula for sampling of the ectocervix. The specimens were placed in liquid solution provided by Cytyc Corporation (Marlborough, MA, USA) and stored at the clinic before being batched-shipped to Cytyc every 2 weeks for immediate processing, analysis and interpretation by a senior, board certified cytologist, according to Bethesda (2001) nomenclature. A total of 10 % of all negative tests and 10 % of all positive tests were re-screened by a senior cytopathologist for purposes of quality assurance.

Immediately after performing the Pap smear, the nurse placed 3–5 % acetic acid on the cervix for 3 min and performed VIA and DC. To obtain the digital cervicographs, a hand-held commercial brand digital camera was used to take a photo of the entire cervix. The image was then magnified to facilitate assessment of acetowhite lesions.

All women also received a DC-directed biopsy with a 2 × 4 mm tip Tischler biopsy forceps. This included a biopsy of the lesion that appeared to have the most advanced degree of neoplasia (such as prominent and opaque acetowhite lesions with sharp borders, coarse punctuations, or atypical blood vessels), as well as a biopsy of a normal appearing area of the transformation zone of the cervix. If the cervix had no abnormal area, only a normal area biopsy within the transformation zone was taken; conversely, if the cervix had no normal area, only an abnormal area biopsy was taken. Biopsy specimens were immediately placed in 10 % formalin and sent to a local pathology laboratory for histopathologic analysis. A combined histology variable was created to represent the most severe diagnosis from the normal and abnormal areas for each woman. Women with confirmed ICC were referred to a specialist hospital in Lusaka for surgery or radiation.

The number of women enrolled into the study was sufficient to give a reasonable estimate of CIN3+. With an expected frequency of 10 %, confidence limits of 5 %, and a 99 % confidence level, the sample size needed is 239 women.

### Number of HIV positive women in Zambia

To estimate the number of HIV positive women aged 20–44 in Zambia, we used the AIDS Impact Module of Spectrum model v.5.03, produced by the Futures Institute (available at: http://www.futuresinstitute.org/spectrum.aspx, accessed April 30, 2014). The AIDS Impact Module projects the number of people living with HIV, new HIV infections, and many other estimates related to the impact of AIDS. Using default Spectrum settings (i.e., generalized epidemic pattern and unchanged female:male incidence ratio), we obtained estimates for the overall number of HIV positive women aged 20–44 in 2013, including stratifications according to antiretroviral therapy (ART) status.

### Prevalence-based model

We constructed a static, prevalence-based model to estimate the number of HIV positive women with CIN3 or ICC in Zambia. The number of HIV positive women in Zambia with CIN3 or ICC was calculated by multiplying the point prevalence of these conditions, based on histology results, from our cross-sectional study by the number of HIV positive women aged 20–44 estimated by Spectrum. To explore the impact of changes in assumptions, we performed several sensitivity analyses. These included using the lower and upper 95 % confidence intervals bounds of CIN3 and ICC prevalence (calculated using the Wilson score method without continuity correction) and adjusting the estimate of HIV positive women with CIN3 or ICC by stratifying by ART status.

### Data analysis

Data cleaning and analysis was conducted using OpenEpi Epidemiologic Calculator (www.openepi.com), Microsoft Excel (Microsoft Corporation, Redmond, WA, USA), and SAS™ version 9.2 (SAS Institute Inc., Cary, NC, USA). *p* < 0.05 was considered statistically significant.

### Ethics statement

The cross-sectional study was approved by the University of Zambia Biomedical Research Ethics Committee on 12 December 2007 (reference number: 008-002-07) and by the University of Alabama at Birmingham Institutional Review Board on 27 June 2007 (registration number: IRB00000726).

## Results

### Demographic data

We compared demographic characteristics between the women from our cross-sectional study and all HIV positive women who have been screened through the CCPPZ in Lusaka (Table 1). Age, marital status, number of lifetime partners, previous Pap smear screening, age at sexual debut, and gravidity were all very similar. Although significant differences were found in age, previous Pap smear, and gravidity, the significance is likely due to the large number of women in the CCPPZ database, and these differences do not appear clinically relevant. Compared with HIV positive women screened through the CCPPZ, women from our cross-sectional study tended to be poorer, less educated, and less likely to work in the formal or informal sectors. In addition, women in the cross-sectional study were more likely to be ARV-experienced than women in the CCPPZ database.

**Table 1:** Demographics of HIV positive women enrolled in the cross-sectional study and in the CCPPZ database

Variable	HIV+ women in cross-sectional study (*n* = 309)	HIV+ women in CCPPZ database^a^ (*n* = 26,993)	*p*-value^b^
Age (years)	306	20,031	<0.001
Median, IQR	32.0 (27.0, 37.0)	35.0 (29.0, 40.0)	
Education	308	20,692	<0.001
Less than high school	275 (89.3 %)	15,425 (74.5 %)	
High school completed	33 (10.7 %)	5,267 (25.5 %)	
Marital status	154	16,212	0.54
Not married	59 (38.3 %)	6,605 (40.7 %)	
Married	95 (61.7 %)	9,607 (59.3 %)	
Employment	307	19,736	<0.001
Not employed outside the home	93 (30.3 %)	6,093 (30.9 %)	
Formal sector	38 (12.4 %)	4,106 (20.8 %)	
Informal sector	85 (27.7 %)	7,291 (36.9 %)	
Other	91 (29.6 %)	2,246 (11.4 %)	
Monthly household income	306	20,121	<0.001
Less than ZMW 500	209 (68.3 %)	9,770 (48.6 %)	
ZMW 500 or more	97 (31.7 %)	10,351 (51.4 %)	
Number of lifetime partners	304	25,312	0.36
Median, IQR	3.0 (2.0, 4.0)	3.0 (2.0, 4.0)	
Pap smear	306	25,278	0.01
Never	306 (100 %)	24,787 (98.1 %)	
Ever	0 (0 %)	491 (1.9 %)	
Age at sexual debut (years)	309	25,303	0.81
Median, IQR	18.0 (16.0, 19.0)	17.0 (16.0, 19.0)	
Gravidity	294	24,247	0.01
Median, IQR	3.0 (2.0, 4.0)	3.0 (2.0, 5.0)	
ARV status	284	22,896	<0.001
ARV-naïve	41 (14.4 %)	6,175 (27.0 %)	
ARV-experienced	243 (85.6 %)	16,721 (73.0 %)	

^a^as of December 2013

^b^By 2-tailed *χ*^2^ test or 2-tailed Wilcoxon two-sample test, as appropriate

CCPPZ, Cervical Cancer Prevention Program in Zambia

#### Test results

**Fig. 1:**
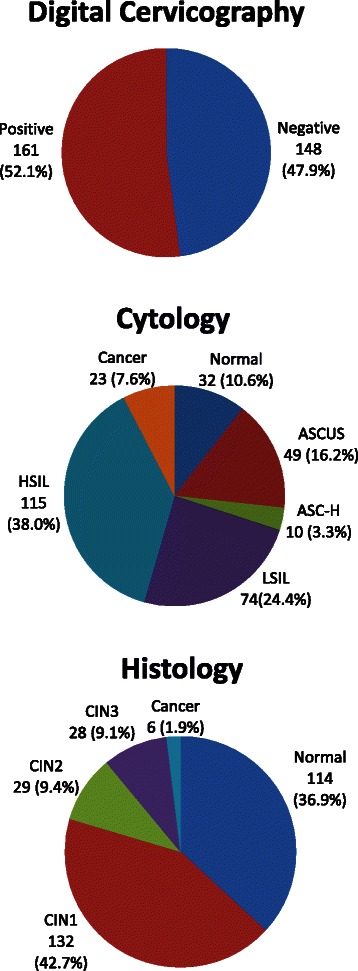
Cervical disease burden by digital cervicography, cytology, and histology. We observed a large cervical disease burden by digital cervicography (52 % positive), cytology (70 % low-grade squamous intraepithelial lesions or worse), and histology (20 % cervical intraepithelial neoplasia 2 or worse). Abbreviations: ASCUS, atypical squamous cells of undetermined significance; ASC-H, atypical squamous cells-cannot exclude HSIL; LSIL, low-grade squamous intraepithelial lesions; HSIL, high-grade squamous intraepithelial lesions; CIN, cervical intraepithelial neoplasia

During DC, the study personnel performed DC-directed biopsy of normal and/or abnormal appearing areas of the cervix. The DC, liquid cytology, and histology results revealed a large cervical disease burden in this previously unscreened HIV positive population of women. Over half (52 %; 161) of the study participants screened positive by DC, while 45 % (138) had cytologic evidence of high grade SIL or worse (HSIL+) (Fig. 1). Histopathologic evaluation revealed that one out of every five (63; 20 %) had evidence of CIN2+ (specifically, 9.4 % had CIN2, 9.1 % had CIN3, and 1.9 % had invasive cancer) (Fig. 1).

### Number of HIV positive women with CIN3 and ICC in Zambia

We used a simple model to estimate the burden of cervical pre-cancer and cancer throughout Zambia. The prevalence of CIN3 in our cross-sectional study of HIV positive women was 9.1 % and the ICC prevalence was 1.9 % (Table 2). The prevalence of both CIN3 and ICC was higher in ART-naïve women than ART-experienced women (Table 2), although this trend was not significant. Using the default parameters of the Spectrum AIDS Impact Module, a total of 375,774 women aged 20–44 are estimated to be HIV positive in Zambia.

**Table 2:** Prevalence of CIN3 and ICC in HIV positive women accessing cervical cancer screening in Lusaka

		CIN3		ICC	
	Total	n	Prevalence (95 % CI)	n	Prevalence (95 % CI)
Overall	309	28	9.1 (6.3, 12.8)	6	1.9 (0.9, 4.2)
ART status					
ART-naïve^a^	41	7	17.1 (8.5, 31.3)	1	2.4 (0.4, 12.6)
ART-experienced	243	18	7.4 (4.7, 11.4)	4	1.6 (0.6, 4.2)
Unknown	25	3	12.0 (4.2, 30.0)	1	4.0 (0.7, 20.0)

^a^The prevalence of CIN3 and ICC is not statistically different between ART-naïve women and ART-experienced women

95 % CI, 95 % confidence interval; ART, antiretroviral therapy; CIN, cervical intraepithelial neoplasia; ICC, invasive cervical cancer

**Fig. 2:**
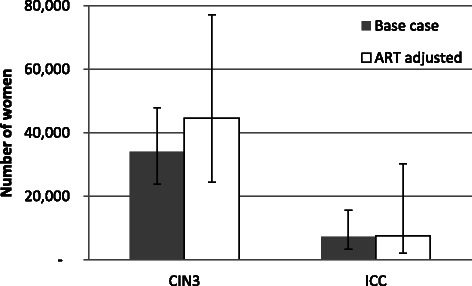
Estimated number of HIV positive women aged 20–44 with cervical intraepithelial neoplasia 3 (CIN3) and invasive cervical cancer (ICC) in Zambia. Among HIV positive women, approximately 34,000 have CIN3 and 7,300 have ICC in the base case scenario. When adjusted for ART status, the estimated numbers of women with CIN and ICC increase. Bars depict 95 % confidence intervals. Abbreviations: ART, antiretroviral treatment

In the base case scenario, we estimated that there are 34,051 HIV positive women with CIN3 in Zambia (Fig. 2). Using the lower and upper 95 % confidence intervals of CIN3 prevalence from the cross-sectional study, the estimates ranged from 23,824 to 48,024 women, and adjusting the number of HIV positive women with CIN3 by ART status (by stratifying by ART status) yielded an estimate of 44,576. The ART-adjusted estimate is higher than the base case because there is a higher prevalence of CIN3 in ART-naïve women (Table 2) and a higher proportion of ART-naïve women from the Spectrum model than in our cross-sectional study in Lusaka. In the base case scenario, we estimated that there are 7,297 HIV positive women with ICC in Zambia (Fig. 2). The other ICC estimates follow a similar pattern to the CIN3 scenarios, with wide 95 % confidence intervals because of small numbers of women with ICC.

## Discussion

We report a very high burden of cervical neoplasia among a group of previously unscreened HIV positive women, whether measured by DC, cytology, or histology. Half screened positive by DC, almost half had HSIL+ on cytology, and one in five was diagnosed with CIN2+ on histopathologic evaluation. Our cytology results are consistent with the previous study by this group [14], which reported an HSIL+ prevalence of 53 %. In addition, in the current study we found that one in four women had low-grade squamous intraepithelial lesions (LSIL), leading to 70 % of women having LSIL+. Across Zambia, the CIN3 and ICC disease burden among HIV positive women is substantial: we estimate that among the 375,774 HIV positive women in Zambia, 34,051 have CIN3 and 7,297 have invasive cervical cancer.

Our 70 % prevalence of LSIL+ is extremely high, even by sub-Saharan African standards. It is much higher than that reported in Nigeria [10], and somewhat higher than results from Cameroon [11] and the 50 % reported in South Africa [13]. Likewise, the burden as measured by histology in our study is higher than a report from Kenya, which detected CIN1 and above in 27 % of HIV positive women attending an ART visit [12].

A strength of our study is the use of three distinct measurements of cervical disease: DC, cytology, and histology. All three demonstrated a large cervical disease burden in this population. Other strengths are the histology results on which our prevalence estimates are based: all women received a histology diagnosis, and we obtained histology results from both normal and abnormal-appearing areas of the cervix. These aspects mean that our CIN3 and ICC prevalence estimates are not underestimated by only having a histology diagnosis for women who screened positive, and that the prevalence estimates are not underestimated by only sampling abnormal-appearing areas [19, 20].

We note several limitations to our analysis. First, there is a relatively small sample size in the cross-sectional study on which our prevalence estimates are based, including the number of ART-naïve women, which leads to relatively large confidence intervals. Second, in our static model we could not account for inputs that change over time, such as changes in HIV prevalence or CIN3 or ICC prevalence. Dynamic modeling could account for these changes and other aspects, such as new disease or recurring disease after treatment. However, an advantage of our model is that it is straightforward to understand and provides useful information to policymakers on the number of HIV positive women with CIN3 and ICC who require cervical cancer screening and treatment. A third weakness is that, compared with HIV positive women screened through the CCPPZ, women from our cross-sectional study tended to be poorer, less educated, and be less likely to work in the formal or informal sector. Thus, our study population is not entirely representative of all of the women screened through the CCPPZ. However, we performed logistic regression to identify possible demographic variables associated with disease, and found that none of the demographics were significantly associated with CIN2+ or CIN3+ (data not shown). This agrees with the previous study by this group [14] where we found that no demographic variables were associated with cytology results in HIV positive women. Thus, the demographic differences between our cross-sectional study and women screened through the CCPPZ are not likely to substantially influence the prevalence estimates of CIN3 and ICC, although the sample size of this study may have limited the power to detect associations between demographic variables and disease.

The very large burden of cervical disease in HIV positive women reported here necessitates a multi-pronged prevention and control approach, including primary prevention with vaccination, secondary prevention with screening and treatment of precancerous lesions, and treatment of cervical cancers. This group has previously reported that approximately 70 % of cervical cancers at the University Teaching Hospital in Lusaka are positive for HPV-16 and/or HPV-18, the two HR-HPV genotypes in widely available vaccines [21]. In addition, immune responses to HPV vaccination among HIV positive women are reported to be generally robust [22]. Taken together, these data indicate that if widely used, HPV vaccination can play an important role in preventing cervical pre-cancer and cancer in HIV positive women in Zambia in the medium- to long-term.

Affordable screening for cervical cancer is feasible and effective in developing countries [23], and due to the large cervical burden, HIV positive women should be a main focus for screening programs. In 2008, less than 15 % of Zambian women have ever had a pelvic exam [24]. CCPPZ currently includes 33 screening clinics and 22 LEEP clinics; an expansion is planned to include 100 additional screening clinics by December 2016 (G. Parham, personal communication). The current capacity and future expansion of CCPPZ will allow ever-increasing numbers of women access to screening and treatment. However, based on our estimates there is a very large burden of CIN3 and ICC in HIV positive women in Zambia, which requires further expanded support for cervical cancer control from both local stakeholders (such as local government and private companies) and external funders, to maintain the current momentum and continue to radically expand across Zambia, to ensure that all HIV positive women in Zambia have access to cervical cancer screening and treatment. Much greater access to prevention services is needed, with the understanding that they must be sustainable for their impact to endure. Involvement of influential components of civil society (for example, traditional healers, traditional marriage counselors, tribal chiefs and chieftainesses) in the process of community education are warranted to extend cancer prevention messages deeper into the community [25].

Due to late presentation and lack of access to effective treatment, survival from cervical cancer in Africa is poor [26]. Therefore, education to increase awareness of cervical cancer screening and treatment, along with increased availability of radiation, chemotherapy, and surgical oncology treatment, should also be a priority.

Integrated cervical cancer prevention, screening, and treatment in Africa is possible; Rwanda has successfully implemented this, and has also integrated these services into routine women’s health services [27]. Pairing cervical cancer prevention, screening, and treatment with other programs such as HIV screening or treatment programs should lead to improved access to cervical cancer services for HIV positive women in Zambia. Political will, partnerships, and monitoring and evaluation are all needed for this to be successful, but with expanded support for cervical cancer control and adequate planning, high quality service integration can be achieved [27].

## Conclusions

Using DC, cytology, and histologic evaluation of both abnormal and normal appearing areas of the cervix, we have demonstrated the large burden of high-grade cervical neoplasia among previously unscreened HIV positive women in Zambia. The planned expansion of CCPPZ will allow ever-increasing numbers of women to access screening and treatment services; however, the commitment of both local stakeholders and external funders should be rapidly expanded to address the large burden of disease. In light of recent strategies aimed at exponentially accelerating access to ARV’s in sub-Saharan Africa (e.g., Option B+), it is imperative that simultaneous investments are made in scaling up cervical cancer prevention services for this very high risk population, to prevent the tragedy of HIV positive women living long enough to develop and die from invasive cervical cancer. An integrated cervical cancer prevention, screening, and treatment approach is needed, and pairing this with ongoing HIV programs should lead to improved access to cervical cancer services for HIV positive women in Zambia.
